# Intracellular bacterial LPS drives pyroptosis and promotes aggressive phenotype in oral squamous cell carcinoma

**DOI:** 10.1007/s12032-025-02766-6

**Published:** 2025-05-08

**Authors:** Shrabon Hasnat, Marjut Metsäniitty, Katariina Nurmi, Kari K. Eklund, Abdelhakim Salem

**Affiliations:** 1 Department of Oral and Maxillofacial Diseases, Clinicum, Faculty of Medicine, University of Helsinki, 00014 Helsinki, Finland; 2 Translational Immunology Research Program (TRIMM), Research Program Unit (RPU), University of Helsinki, 00014 Helsinki, Finland; 3 Department of Rheumatology, University of Helsinki and Helsinki University Hospital, 00014 Helsinki, Finland; 4 Head and Neck Oncobiome Group, Department of Oral and Maxillofacial Diseases, Clinicum, Faculty of Medicine, University of Helsinki, 00014 Helsinki, Finland

**Keywords:** Oral squamous cell carcinoma, Oral dysplasia, HPV-induced malignant transformation, Lipopolysaccharide, Pyroptosis, Noncanonical inflammasome, Cancer invasion

## Abstract

Intracellular bacterial components represent an emerging tumor element that has recently been documented in multiple cancer types, yet their biological functions remain poorly understood. Oral squamous cell carcinoma (OSCC) is a particularly aggressive malignancy lacking highly effective targeted treatments. Here, we explored the functional significance of intracellular bacterial lipopolysaccharide (LPS) in OSCC. Normal human oral keratinocytes (HOKs), HPV-transformed oral keratinocytes (IHGK), and three OSCC cell lines were transfected with ultrapure bacterial LPS. Cytotoxicity was assessed via lactate dehydrogenase (LDH) release assays. Production of interleukin (IL)-1β and IL-18 was measured using ELISA. Impact on tumor progression was evaluated using cell proliferation, migration, invasion, and tubulogenesis assays. Intracellular LPS-induced significant LDH release and increased secretion of IL-18 and IL-1β in IHGK and cancer cells, but not in normal HOKs, indicating selective cytotoxicity and pyroptosis. Notably, metastatic cancer cells exhibited enhanced invasive and vessel-like structures upon LPS exposure, while IHGK cells exhibited increased proliferation without changes in migration. Our findings suggest that intracellular LPS may not merely reside passively within the tumor milieu, but could contribute to OSCC progression by triggering noncanonical inflammasome activation and pyroptosis. This process may enhance pro-inflammatory signaling and more aggressive cellular phenotypes, especially in metastatic settings. Targeting intracellular LPS or its downstream inflammasome pathways may thus represent a promising therapeutic strategy for OSCC, warranting further in vivo and clinical investigations.

**Supplementary Information:**

The online version contains supplementary material available at 10.1007/s12032-025-02766-6.

## Introduction

Oral squamous cell carcinoma (OSCC) stands as the most prevalent malignancy within the head and neck tissues, posing a global health burden. According to the latest GLOBOCAN report, approximately 390,000 new cases and an estimated 188,000 deaths due to lip and oral cavity cancer were recorded worldwide in 2022 [1]. Despite advancements in diagnostic and therapeutic strategies, the 5 year survival rate for OSCC patients hovers around 60% in many countries, including Finland and Sweden [2]. Traditional risk factors such as tobacco use, excessive alcohol consumption, and human papillomavirus (HPV) infection have been well-documented [3, 4]. However, a significant proportion of OSCC cases develop from existing oral potentially malignant disorders (OPMDs), such as oral leukoplakia, which are often associated with dysplastic changes and chronic inflammation, underscoring the complex etiology of this disease [5].

Emerging evidence highlights the critical interplay between the oral microbiota, particularly bacteria, and OSCC pathogenesis, where dysbiosis may drive chronic inflammation and tumor-promoting mechanisms [6]. Recent studies have reported an increased prevalence of Gram-negative bacteria such as *Fusobacterium* and *Porphyromonas* species, alongside a decreased presence of Gram-positive *Streptococcus* species, in both OSCC and OPMD tissues [6, 7]. Anaerobic bacteria associated with periodontitis have been linked not only to OSCC [8–10], but also to other malignancies such as lung cancer [11] and hematopoietic cancers [12]. A comprehensive meta-analysis by Corbella et al. [13] further established a significant association between periodontal disease and various cancers, including those of the digestive tract, pancreas, prostate, breast, and lungs, as well as non-Hodgkin lymphoma. However, the intricate interactions between oral bacteria and cancer cells remain largely unexplored, necessitating deeper investigation into the microbial contributions to oral carcinogenesis.

Lipopolysaccharide (LPS), a key component of the outer membrane of Gram-negative bacteria, plays a pivotal role in host-microbe interactions. As a potent proinflammatory molecule, extracellular LPS is known to activate canonical inflammatory responses through Toll-like receptors (TLR2 and TLR4) expressed on the surface of various cell types, including epithelial cells [14]. In addition to these surface pathways, LPS can also engage intracellular innate immune receptors. Intracellular delivery of LPS may occur via multiple mechanisms, including bacterial outer membrane vesicles (OMVs), in which LPS is one of the most abundant components, endocytosed host-derived extracellular vesicles, or association with carrier proteins such as high mobility group box 1 protein [15–17]. In the context of cancer, microbial access to deeper tissue compartments may be facilitated by disruption of the epithelial barrier and direct interaction with invading tumor cells [18, 19]. Once inside the host cell, intracellular LPS activates the noncanonical inflammasome pathway, leading to pyroptosis—a form of lytic cell death—and the secretion of potent pro-inflammatory cytokines like interleukin-18 (IL-18) and interleukin-1β (IL-1β). This process is accompanied by the release of damage-associated molecular patterns (DAMPs) and extracellular vesicles, which activate the immune system eliciting strong inflammatory responses [20, 21]. While this mechanism serves as a critical defense against microbial pathogens, uncontrolled inflammation has been implicated in the development of chronic diseases, including cancer [21]. These dual modes of LPS signaling—extracellular and intracellular—are highly relevant for understanding its multifaceted role in shaping tumor behavior.

In recent years, growing evidence has revealed the presence of microbiota as integral components of tumor tissues across a wide range of cancers, including those previously considered sterile, such as pancreatic, lung, and breast cancers [22–25]. In their comprehensive analysis of over 1500 solid tumor samples from multiple cancer types, Nejman et al. [24] discovered that tumors harbor distinct communities of intracellular bacteria. These bacteria were found to be tumor-type specific, residing within both cancer and immune cells. Notably, the presence of these intracellular bacteria correlated with patients’ responses to immunotherapy, suggesting that the tumor microbiome may influence therapeutic outcomes. These findings imply that intracellular bacterial components, such as LPS, could play a significant role in tumor biology and potentially affect the progression of OSCC. Despite established links between bacteria and cancer, the specific role of intracellular LPS in modulating OSCC progression remains largely uncharted.

## Materials and methods

### Cell lines and culture conditions

The following cell lines were utilized: Cancer cell lines including the highly invasive HSC-3 (Japan Health Sciences Foundation, Tokyo, Japan), UT-SCC-24 A (primary tongue cancer) and UT-SCC-24B (metastatic tongue cancer) from Turku University Hospital (hereafter SCC-24 A and −24B, respectively); IHGK cells (HPV-16-transformed oral keratinocytes with molecular premalignant features as a model of early epithelial malignant transformation; [26]),and normal human oral keratinocytes (HOKs; ScienCell Research Laboratory, Carlsbad, CA, USA). Cancer cells were cultured in Dulbecco’s modified eagle medium (DMEM/F-12, Gibco, 31,330–038, Waltham, USA) supplemented with 10% heat-activated fetal bovine serum (Gibco, 10,270–106), 50 µg/ml ascorbic acid, 100 U/ml penicillin, 100 µg/ml streptomycin, 250 ng/ml fungizone and 0.4 µg/ml hydrocortisone (all from Sigma-Aldrich, St. Louis, USA). IHGK cells were maintained in Keratinocyte-SFM Medium (Gibco 17,005–075) with L-glutamine, epidermal growth factor, and bovine pituitary extract, supplemented with 100 U/ml penicillin, 100 µg/ml streptomycin, 250 ng/ml fungizone and 50 µl calcium chloride. HOKs were cultured in Oral Keratinocyte Medium (ScienCell). All cells were incubated at 37 ℃ in a humidified atmosphere with 5% CO_2_. Cell lysates were collected and stored at − 80 ℃ until further use.

### Transfection of ultrapure LPS

Ultrapure LPS from *Escherichia coli* (O111:B4, 2 µg/ml; InvivoGen, San Diego, CA, USA) was transfected into cells using Lipofectamine 2000 (5 µl; LFA) reagent according to the manufacturer’s protocol (Invitrogen, Thermo Fisher Scientific, Waltham, MA, USA). Briefly, cells were seeded on 24-well plates and cultured until 70–80% confluency. Upon reaching confluence, the culture medium was replaced with serum-free Opti-MEM™ medium (Gibco), and all stimulations and transfections were performed under these standardized conditions. LFA was diluted in Opti-MEM™ and mixed with LPS, and the mixture was incubated for 20 min to allow the complex formation. The LFA/LPS complexes were then added to the cells and incubated for 6, 18, or 30 h. Supernatants from untreated cells (control groups), cells treated with LFA alone (mock transfection), LPS alone (extracellular LPS), and LFA/LPS-transfected cells (intracellular LPS) were collected, centrifuged to remove debris, and stored at –80 ℃ for subsequent experiments. In the extracellular LPS condition, ultrapure LPS was diluted in the serum-free medium and added directly to the cells to mimic physiological exposure conditions.

### Cytotoxicity assay

Cytotoxicity was assessed by measuring lactate dehydrogenase (LDH) release using the Cytotoxicity Detection Kit (Roche Diagnostics, Mannheim, Germany; Cat. No. 11644793001). Cells were seeded in 96-well plates and transfected with LPS as described above. After incubation, supernatants were collected, and LDH activity was measured spectrophotometrically at 490 nm using a microplate reader (BioTek Instruments, Winooski, VT, USA). To normalize cytotoxicity, maximum LDH release was determined by lysing parallel control wells with 1% Triton X-100 for 10 min at room temperature, following standard protocol as recommended by manufacturer. Cytotoxicity was then calculated as percent change relative to maximum LDH release.

### Enzyme-linked immunosorbent assay (ELISA)

The concentrations of total IL-18 and mature IL-1β in cell culture supernatants were quantified using DuoSet ELISA Development Systems (R&D Systems, Minneapolis, MN, USA) according to the manufacturer’s instructions. Equal confluency was confirmed microscopically across all wells prior to stimulation. Color development was achieved using tetramethylbenzidine (TMB) substrate and absorbance was analyzed with microplate reader at 450 nm with correction at 540 nm.

### Transwell invasion and migration assays

Transwell assays were performed using 24-well inserts with 8 μm pore membranes (Corning Inc., Corning, NY, USA), as recently described [8]. For invasion assays, the inserts were coated with a myogel matrix supplemented with 0.8 mg/ml type I rat tail collagen (Sigma-Aldrich, St. Louis, MO, USA) to simulate the extracellular matrix. HSC-3, SCC-24 A, and SCC-24B cancer cells were seeded onto the coated inserts at a density of 70 × 10^3^ cells per insert and treated with the pyroptotic supernatants or other individual treatments for 72 h.

Migration assays were conducted using uncoated Transwell inserts to assess cell movement independent of matrix invasion. IHGK cells were seeded at a density of 70 × 10^3^ cells per insert in the upper chambers, while pyroptotic supernatants were added to the lower chambers as chemoattractants. Migration was allowed for 48 h.

Following incubation, cells were fixed with 4% neutral-buffered formalin for 1 h and washed with PBS. The inserts were then stained with 1% Toluidine Blue in 1% Borax for 10 min at room temperature and rinsed thoroughly with distilled water. Non-invaded (or non-migrated) cells remaining on the upper membrane surface were gently removed using a moistened cotton swab. To quantify cell invasion or migration, the retained dye was eluted using 1% SDS, and absorbance was measured at 650 nm using a microplate reader.

### IncuCyte proliferation assay

Proliferation of IHGK cells were monitored using the IncuCyte® Live-Cell Analysis System (Sartorius, Göttingen, Germany). Cells were labeled with CellTrace™ Far Red dye (Thermo Fisher Scientific, Waltham, MA, USA) following the manufacturer’s protocol. Dye-labeled cells were seeded at a density of 4 × 10^3^ cells per well in a 96-well plate (Corning, Inc., Corning, NY, USA), and allowed to adhere overnight. The next day, media were replaced with the respective pyroptotic supernatants and other individual treatments. Real-time imaging was conducted every 2 h over 72 h. Proliferation was assessed by quantifying the fluorescence intensity of the CellTrace dye.

### Tumor cell tubulogenesis assay

To evaluate the angiogenic potential of the highly invasive cancer cells, tube formation assays were performed using Matrigel® Basement Membrane Matrix (Corning, Inc., Corning, NY, USA). Briefly, 24-well plates were coated with 100 μl of Matrigel per well and incubated at 37 ℃ for 30 min to solidify. HSC-3 and SCC-24B cells were pre-treated with their respective pyroptotic supernatants and seeded at 1 × 10^5^ cells per well. Control cells were incubated with conditioned media from untreated cells. Cells were incubated at 37 ℃, and the tube formation was imaged at 4-, 8-, and 18 h intervals using a Carl Zeiss Primo Vert inverted microscope equipped with an AxioCam ERc5 s camera (Zeiss, Oberkochen, Germany). The ImageJ software with “Angiogenesis Analyzer” plugin was utilized to analyze the tube formation (Wayne Rasband, National Institute of Health, Bethesda, MD, USA).

### Statistical analysis

Statistical analyses were conducted using GraphPad Prism software version 10.1 (GraphPad Software, San Diego, CA, United States). Data are presented as mean ± standard error of the mean (SEM) from three independent experiments in duplicates. One-way or Two-way analysis of variance, when applicable, followed by Tukey’s multiple comparison test was used to determine statistical significance among groups. ^*^ *P* ≤ 0.05; ^**^ *P* ≤ 0.01; ^***^ *P* ≤ 0.001; ^****^ *P* ≤ 0.0001.

## Results

### Intracellular LPS induces variable LDH release in HPV-transformed and cancer cells

First, we investigated whether intracellular LPS could induce cytotoxic changes in normal (HOKs) and HPV-transformed (IHGK) oral keratinocytes, as well as in OSCC cell lines. To this end, cells were treated with LFA, LPS, or transfected with a complex of LFA/LPS, and LDH release was measured at 6, 18, and 30 h. To specifically assess the effects of intracellular LPS without confounding factors, we utilized ultrapure LPS from *E. coli* O111:B4. This ultrapure form is devoid of contaminating bacterial components (e.g., lipoproteins and nucleic acids), ensuring that the observed cellular responses are attributable solely to LPS. Ultrapure LPS is widely used in studies of inflammasome activation and pyroptosis model [27], making it an ideal choice in our cellular assays.

**Fig. 1:**
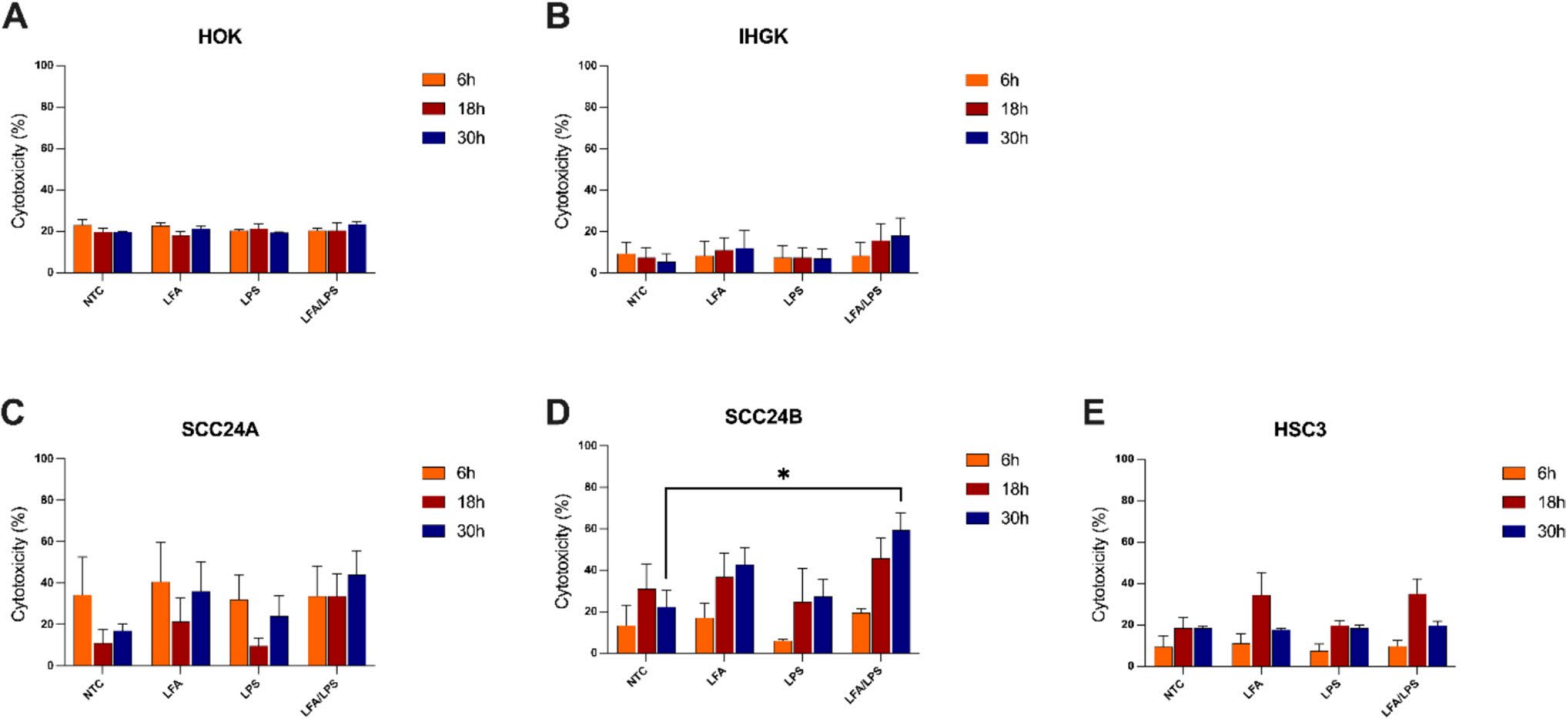
Effect of intracellular LPS on lytic cell death across oral epithelial cell lines. Cells were transfected with ultrapure LPS from *Escherichia coli* O111:B4 (2 µg/ml) using Lipofectamine 2000 (LFA/LPS) or with either LFA or LPS diluted directly in the medium. Treatments were performed for 6, 18, and 30 h, followed by analysis of LDH release. **A** Normal human oral keratinocytes (HOKs) showed minimal LDH release in response to intracellular LPS. In contrast, LPS transfection led to non-significantly increased LDH release in **B** HPV-transformed IHGK cells and (**C**–**E**) cancer cell lines, with SCC-24B exhibiting a statistically significant increase in response to LFA/LPS treatment at 30 h. ^*^P < 0.05. NTC: No-treatment control. Data represent mean ± SEM. Experiments were repeated independently three times with duplicates for each condition

In HOKs, LPS transfection (hereafter intracellular LPS) did not noticeably affect LDH release at any time point (Fig. 1A), suggesting that normal oral epithelial cells may exhibit resistance to LPS-induced cytotoxicity under these conditions. In contrast, LPS transfection increased LDH release in IHGK and OSCC cell lines, signifying enhanced lytic changes (Fig. 1B–E). This increase was statistically significant in SCC-24B cells (*P* < 0.05; Fig. 1D), while IHGK, SCC-24 A and HSC-3 cells showed slight nonsignificant (*P* = 0.50, 0.42 and 0.99, respectively) elevation of LDH secretion. Moreover, intracellular LPS-induced a markedly stronger cytotoxic response compared to extracellular LPS, which had no observable effect in IHGK and OSCC cell lines (Fig. 1B–E).

### Intracellular LPS elicits production of pyroptotic cytokines in HPV-transformed and cancer cells

**Fig. 2:**
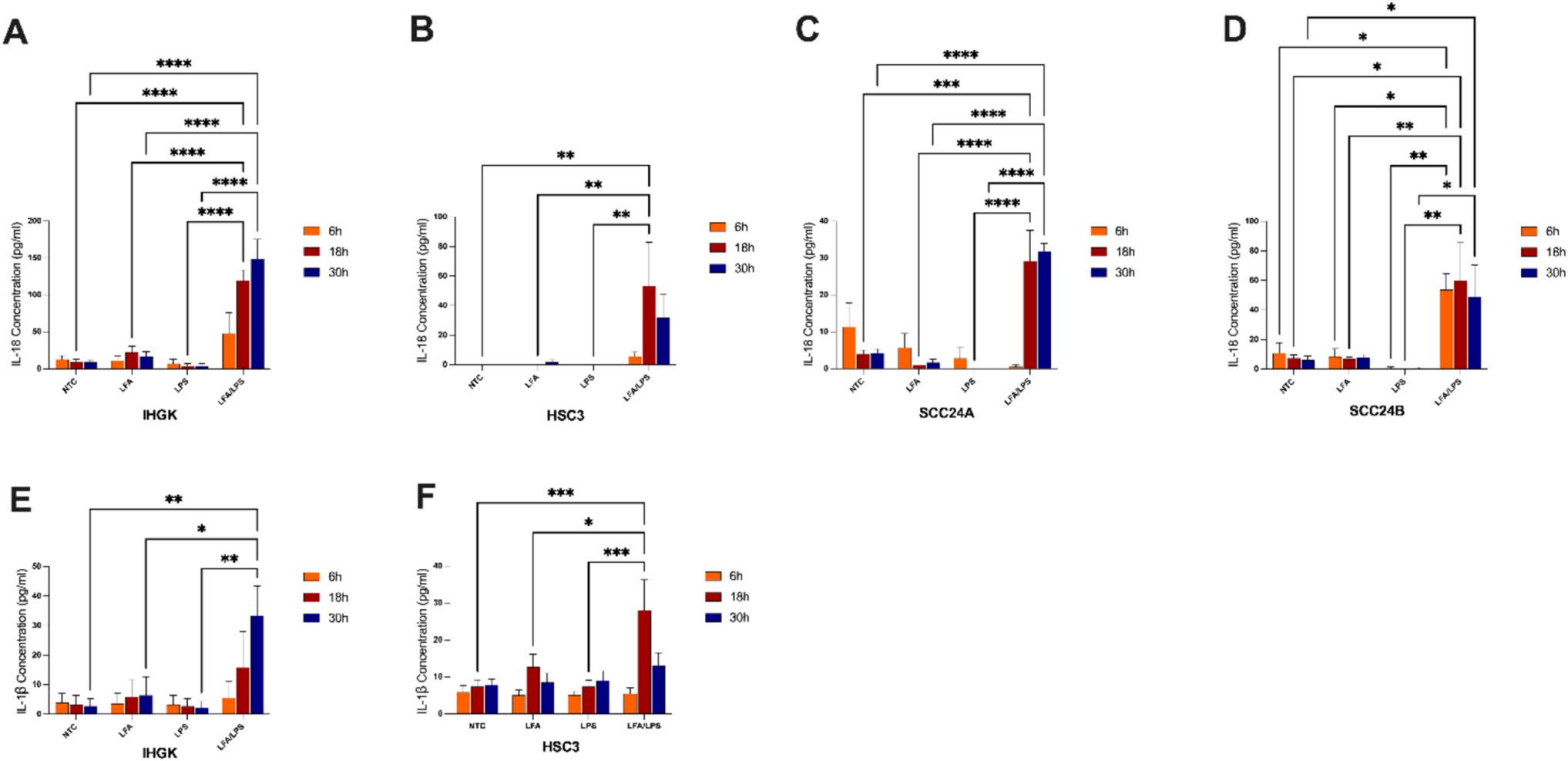
Effect of intracellular LPS on the production of pyroptotic cytokines across oral epithelial cell lines. Cells were transfected with ultrapure LPS from *Escherichia coli* O111:B4 (2 µg/ml) using Lipofectamine 2000 (LFA), or with either LFA or LPS diluted directly in the medium for the indicated times to assess the noncanonical inflammasome activation by measuring IL-18 and IL-1β secretion. **A**–**D** IL-18 secretion was significantly elevated in response to intracellular LPS in HPV-transformed IHGH cells, as well as in cancer cell lines: HSC-3 (**B**), SCC-24 A (**C**) and SCC-24B (**D**) compared to the individual treatments and untreated controls. **E**–**H** IL-1β secretion was also notably increased in IHGK cells (**E**) and HSC-3 cells (**F**). ^**^P < 0.01, ^***^ P < 0.001, ^****^ P < 0.0001. NTC: No-treatment control. Data represent mean ± SEM. Experiments were repeated independently three times with duplicates for each condition

Given the observed cytotoxicity, we next assessed whether intracellular LPS activates the noncanonical inflammasome pathway in these cells. Noncanonical inflammasome activation triggers pyroptosis and activation of the NLRP3 inflammasome, which in turn cleaveses pro-IL-1β and pro-IL-18 into their mature secreted form, and cell membrane rupture during pyroptosis mediates the bulk secretion of the signature cytokines [28]. LPS transfection substantially increased IL-18 secretion in both IHGK (*P* < 0.0001; Fig. 2A) and OSCC cells: HSC-3 (*P* < 0.01; Fig. 2B), SCC-24 A (*P* < 0.001; Fig. 2C), and SCC-24B (*P* < 0.05; Fig. 2D). Across all cell lines, LPS transfection (LFA/LPS) led to significantly higher IL-18 secretion. Similarly, secretion of another NLRP3 inflammasome-dependent cytokine, IL-1β, was notably elevated in the IHGK cells (*P* < 0.01; Fig. 2E) and HSC-3 cells (*P* < 0.001; Fig. 2F), while SCC-24 A and −24B cancer cell lines showed negligible IL-1β response (*P* > 0.05; Supplementary Fig. 1). In contrast, extracellular LPS had no effect on the secretion of neither IL-18 nor IL-1β. The elevated production of IL-18 and IL-1β in IHGK and OSCC cells reinforces the role of inflammasome activation in promoting a pro-inflammatory microenvironment.

### Intracellular LPS potentiates invasion in metastatic cancer cells

**Fig. 3:**
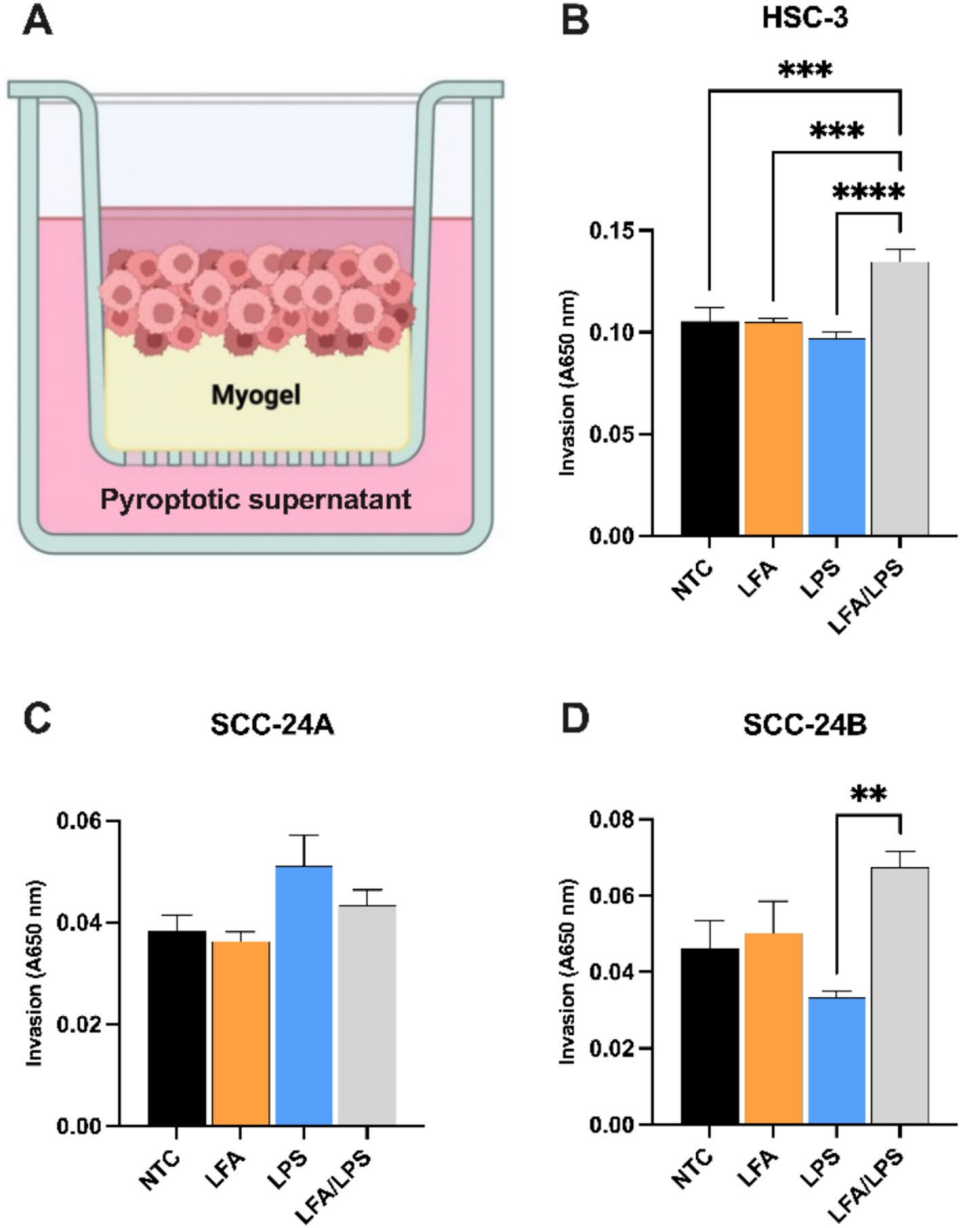
Effects of intracellular LPS-induced pyroptotic supernatants on invasion of primary and metastatic cancer cells. **A** Schematic representation of the Transwell invasion assay using myogel-coated inserts, showing incubation of cancer cells with their respective supernatants collected from untreated cells (control groups), cells treated with Lipofectamine (LFA), ultrapure LPS from *Escherichia coli* O111:B4 (2 µg/ml; i.e., extracellular LPS), and LFA/LPS (pyroptotic supernatants), for 72 h.** B** In HSC-3 cells, invasion significantly increased in response to supernatants collected following LPS transfection (LFA/LPS) compared to individual treatments and untreated controls. **C** SCC-24 A cells showed no significant change in invasion upon exposure to the pyroptotic supernatants. **D** SCC-24B cells demonstrated a significant increase in invasion with supernatants from LPS-transfected cells compared to extracellular LPS alone, though this difference was not statistically significant when compared to untreated controls. ^**^P < 0.01, ^***^ P < 0.001, ^****^ P < 0.0001. NTC: No-treatment control. Data represent mean ± SEM. Experiments were repeated independently three times with duplicates for each condition

To determine whether the pro-inflammatory response elicited by intracellular LPS translates into increased invasive behavior, we performed Transwell invasion assays using myogel-coated inserts. Primary (SCC-24 A) and metastatic cancer cells (SCC-24B and HSC-3) were incubated with their respective pyroptotic supernatants and other individual treatments collected 18 h post-LPS transfection (Fig. 3A). In metastatic HSC-3 cells, exposure to pyroptotic supernatants collected from LPS-transfected HSC-3 cells led to a significantly increased invasion (*P* < 0.001; Fig. 3B), suggesting that upon noncanonical inflammasome activation cells secrete inflammatory signals, which increase invasive capability of the neighboring cells. Similarly, SCC-24B cells showed a significant increase in invasion when treated with supernatant from LPS-transfected SCC-24B cells compared to extracellular LPS treatment (*P* < 0.01; Fig. 3D), though this effect was not statistically significant when compared to untreated controls (*P* > 0.05). Conversely, primary SCC-24 A cells did not show any significant changes in invasion upon exposure to supernatants from LPS-transfected cells (*P* > 0.05; Fig. 3C). These findings suggest that intracellular LPS may preferentially enhance the invasive potential of metastatic over primary cancer cells.

**Fig. 4:**
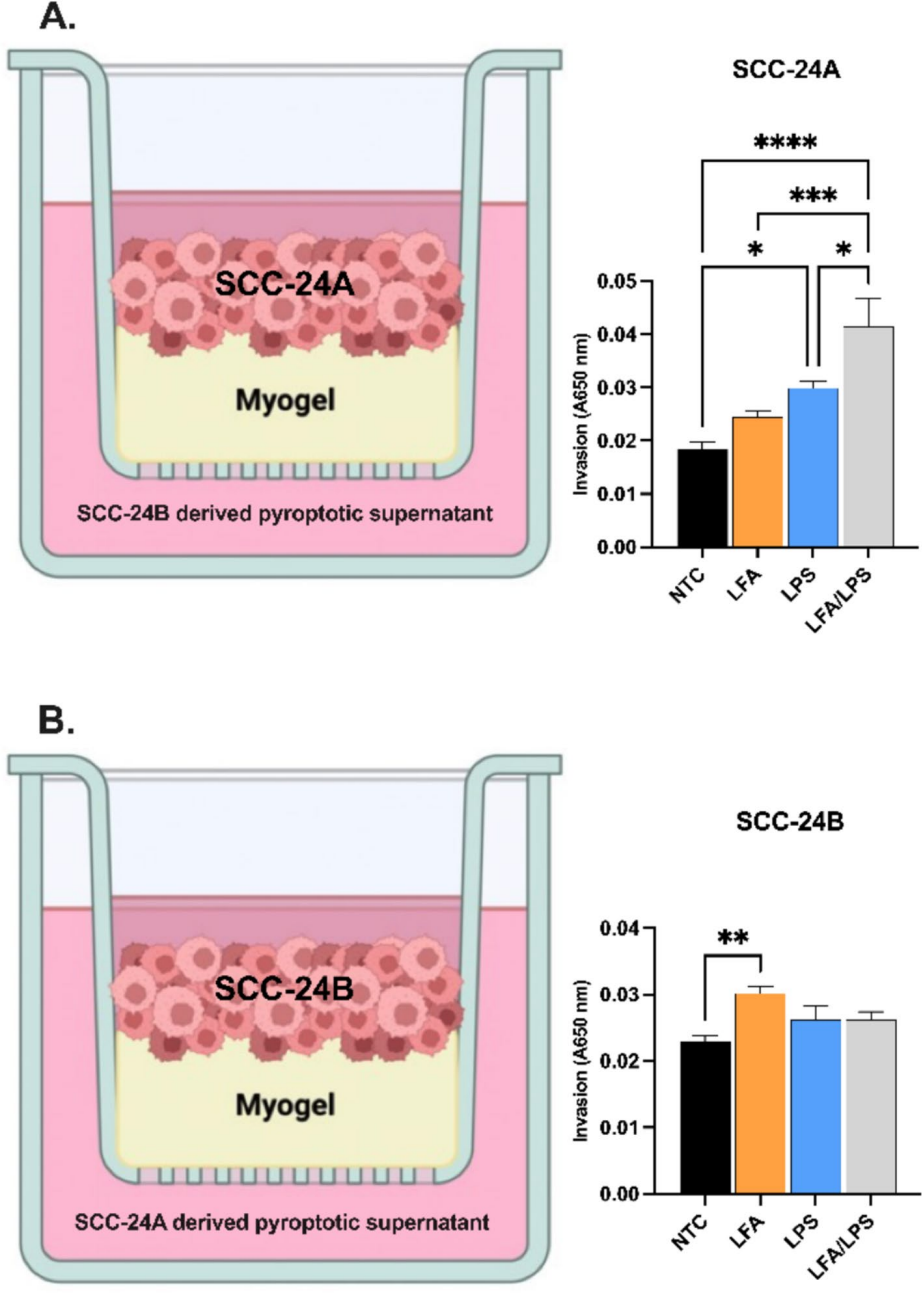
Paracrine effects of pyroptotic supernatants from metastatic and primary cancer cells on invasion.** A** Schematic representation of SCC-24 A cells exposed to pyroptotic supernatants collected from LPS-transfected SCC-24B cells (ultrapure LPS from *Escherichia coli* O111:B4; 2 µg/ml) in the Transwell invasion assay for 72 h, showing a substantial increase in the invasive capacity of SCC-24 A cells compared to individual treatments and untreated controls. **B** SCC-24B cells exposed to pyroptotic supernatants from LPS-transfected SCC-24 A cells did not exhibit a significant increase in invasion, suggesting an asymmetric paracrine effect. ^*^P < 0.05, ^**^P < 0.01, ^***^P < 0.001, ^****^P < 0.0001. NTC: No-treatment control. Data represent mean ± SEM. Experiments were repeated independently three times with duplicates for each condition

To further explore the paracrine effects of noncanonical inflammasome activation, we examined whether pyroptotic supernatants from LPS-transfected metastatic cells could augment the invasive potential of adjacent primary cancer cells. When the primary tumor cells, SCC-24 A, were exposed to pyroptotic supernatants derived from metastatic SCC-24B cells, they exhibited a substantial increase in invasion compared to untreated controls (*P* < 0.0001; Fig. 4A). In contrast, metastatic SCC-24B cells showed no significant change in invasion when exposed to pyroptotic supernatants from their primary counterpart, SCC-24 A (*P* > 0.05; Fig. 4B). This indicates that factors released from metastatic cells stimulated with intracellular LPS may foster a more aggressive phenotype in primary tumor cells, potentially through modulation of the tumor microenvironment (TME).

### Intracellular LPS enhances the proliferation of HPV-transformed oral keratinocytes

**Fig. 5:**
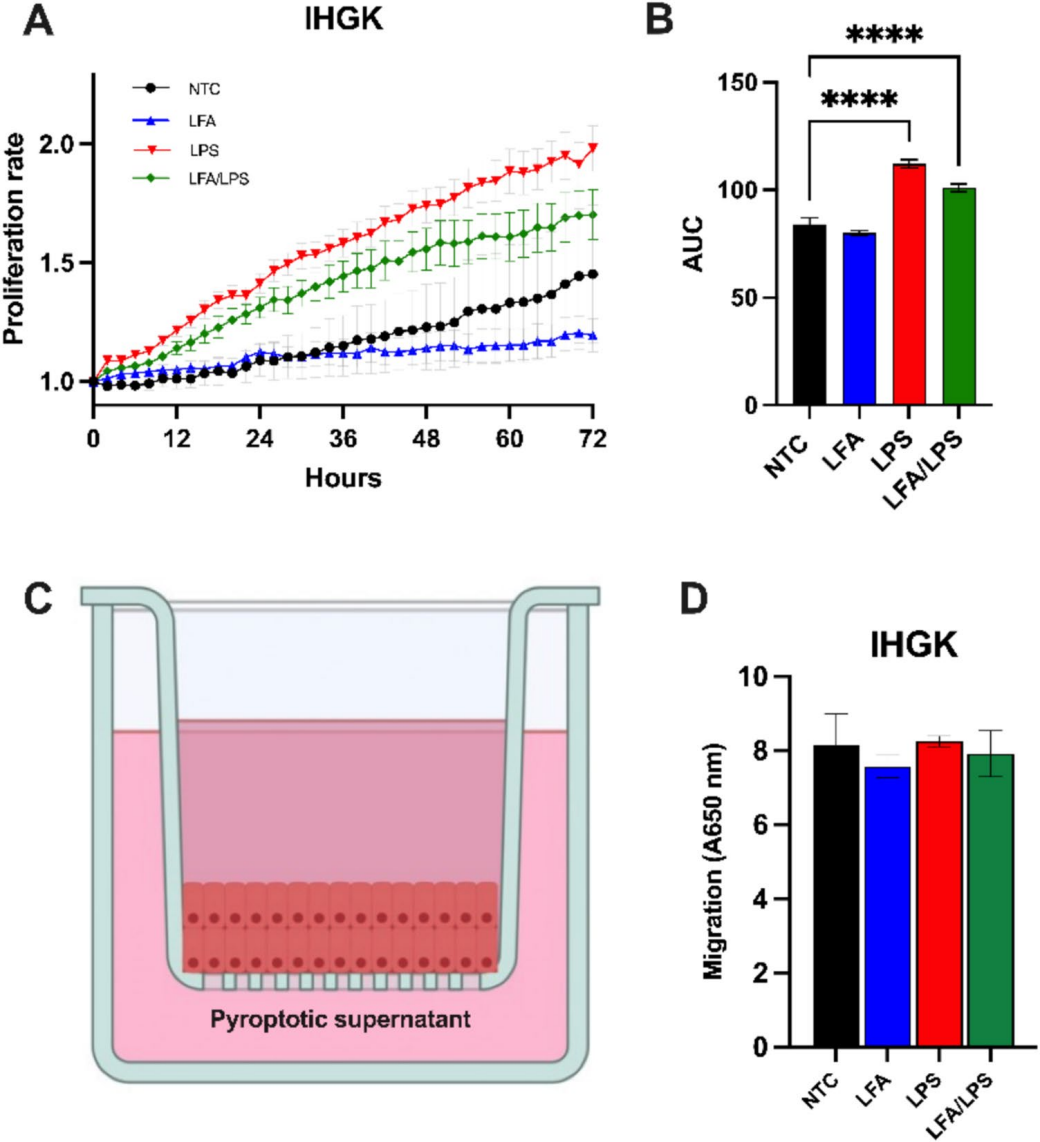
Proliferation and migration effects of intracellular LPS on HPV-transformed oral keratinocytes. **A**, **B** CellTrace™ Far Red dye-labeled IHGK cells were challenged with their respective supernatants collected from untreated IHGK cells (control groups), cells treated with Lipofectamine (LFA), ultrapure LPS from *Escherichia coli* O111:B4 (2 µg/ml; i.e., extracellular LPS), and LFA/LPS (pyroptotic supernatants). Proliferation was assessed by quantifying CellTrace fluorescence intensity over 72 h. HPV-transformed IHGK cells treated with supernatants from LPS-activated cells (extracellular LPS and pyroptotic supernatants) exhibited a significantly higher proliferation rate compared to the controls. The right panel shows the area under the curve (AUC) quantification for proliferation across treatments. **C** Schematic representation of the migration assay setup with IHGK cells and the corresponding quantification of migration. Migration was quantified by measuring the absorbance and it was performed similarly to the invasion assays but without the myogel matrix. **D** No significant increase in migration was observed in IHGK cells, indicating that while intracellular LPS can enhance proliferation, it does not affect the migration capacity of these cells, consistent with the noninvasive characteristics of pre-malignant lesions. ****p < 0.0001. NTC: No-treatment control. Data represent mean ± SEM. Experiments were repeated independently three times with duplicates for each condition

Dysplastic and premalignant epithelial changes are typically incapable of invasion, as this is a hallmark of malignancy. Therefore, we conducted proliferation and migration assays to assess whether the noncanonical inflammasome activation can induce pro-tumorigenic behavior in HPV-transformed oral keratinocytes. IHGK cells were incubated with their respective supernatants including control supernatants from untreated cells and monitored over three days. Our data showed that exposure to supernatants from extracellular LPS- and LFA/LPS-activated IHGK cells exhibited a significantly higher proliferation rate compared to untreated controls (*P* < 0.0001; Fig. 5A). However, neither extracellular nor intracellular LPS significantly affected the migration capacity of these cells (*P* > 0.05; Fig. 5B). These findings suggest that while noncanonical inflammasome activation can stimulate the proliferation of HPV-transformed cells, it does not enhance their migratory potential, consistent with the non-invasive nature of pre-malignant epithelial changes.

### Intracellular LPS promotes robust tubulogenesis in metastatic cancer cells

Given the enhanced invasive potential observed in metastatic cancer cells following noncanonical inflammasome activation, we further examined whether intracellular LPS influences tumor phenotypic plasticity, particularly tubulogenesis—a hallmark of vasculogenic mimicry (VM) that is associated with aggressive tumor phenotype [29]. Metastatic HSC-3 and SCC-24B cells were cultured on Matrigel-coated plates and incubated with their respective pyroptotic supernatants for up to 18 h.

**Fig. 6:**
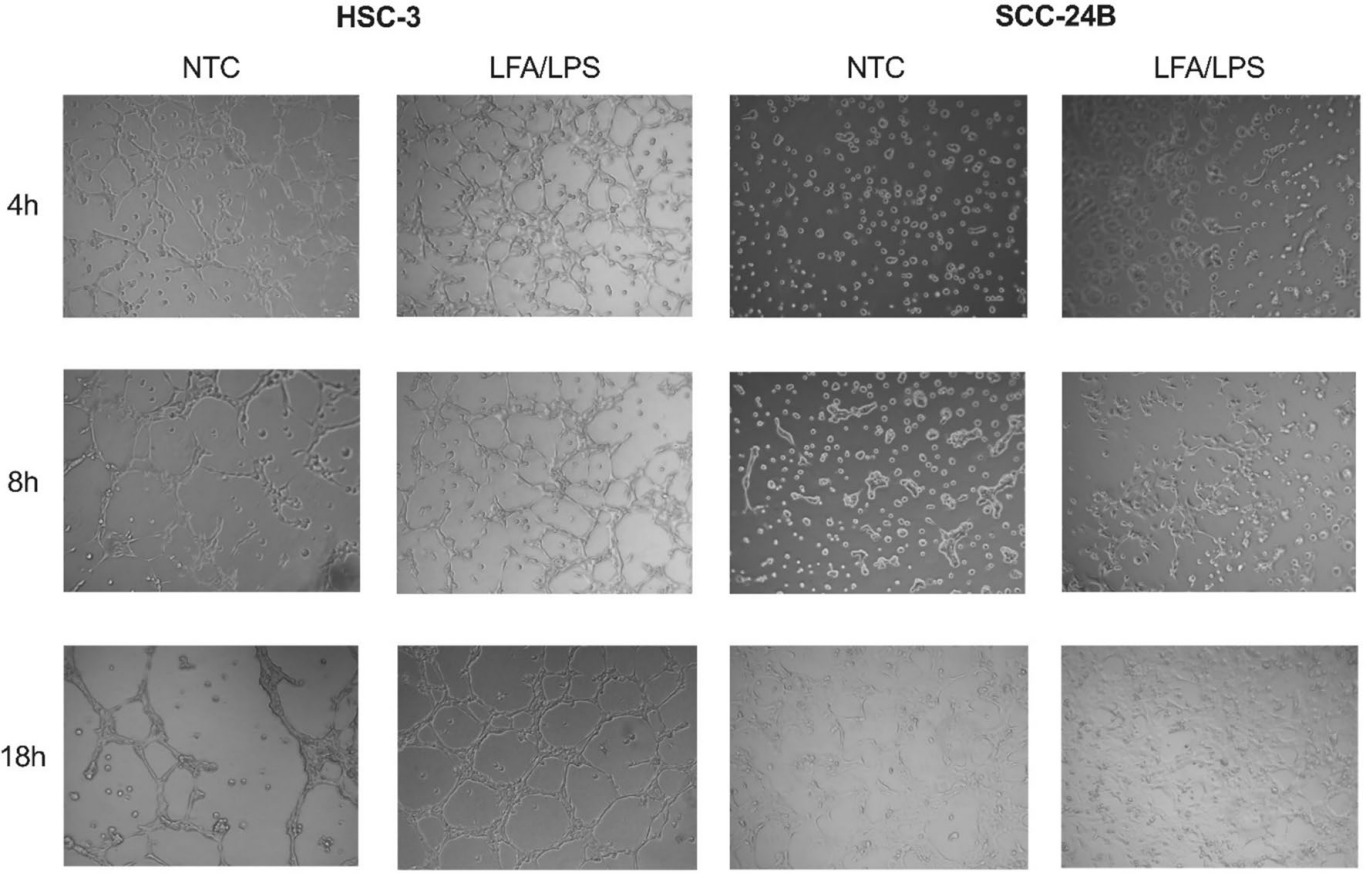
Vessel-like structures induced by intracellular LPS in metastatic cancer cells. Metastatic HSC-3 and SCC-24B cells were cultured on Matrigel-coated plates and exposed to their respective pyroptotic supernatants collected from untreated cells (control groups), cells treated with Lipofectamine and ultrapure LPS (2 µg/ml) from *Escherichia coli* O111:B4 (LFA/LPS; i.e., pyroptotic supernatants) over 18 h to assess tubulogenesis—a characteristic associated with aggressive tumor behavior. Representative images showing that cancer cell treatment with pyroptotic supernatants resulted in a pronounced tubular network formation, especially evident at 8 and 18 h, suggesting that intracellular LPS may enhance tumor phenotypic plasticity and aggressiveness through induction of cancer cell-derived tube formation. *NTC* no-treatment control. Experiments were repeated independently three times with duplicates for each condition

Our results revealed that exposure to pyroptotic supernatants markedly induced the formation of vessel-like structures suggestive of tubular morphology in both HSC-3 and SCC-24B cells compared to untreated controls (Fig. 6). This effect was most pronounced at the 8- and 18 h time points, with extensive tubular network structures forming in treatment groups, suggesting a potential role for intracellular LPS in enhancing tumor aggressiveness through modulation of phenotypic plasticity.

## Discussion

The role of oral microbiota and human carcinogenesis has garnered increasing attention, with accumulating evidence linking Gram-negative bacteria to the development of OPMD and OSCC [6, 8, 30] as well as to proinflammatory bacteriomes [31]. LPS is the major immunomodulatory component of Gram-negative bacteria, activates not only extracellular pattern recognition receptors but also intracellular sensors, notably the noncanonical inflammasome. Although Gram-negative bacteria typically reside extracellularly, intratumoral bacteria in human cancers were predominantly detected in the cytoplasm of both immune cells and tumor cells [24], and their OMVs can deliver LPS into cells [28]. Our current study focused on delineating how intracellular LPS influences OSCC progression.

Our in vitro assays revealed that intracellular LPS-induced selectively lytic cell death in HPV-transformed and OSCC cells, evidenced by increased LDH release. This pyroptotic cell death was accompanied by the other hallmark feature of noncanonical inflammasome activation—elevated secretion of pro-inflammatory cytokines IL-18 and IL-1β. Interestingly, healthy oral epithelial cells did not exhibit significant LDH release in response to LPS transfection, suggesting an increased vulnerability of HPV-transformed and OSCC cells to noncanonical inflammasome-induced pyroptosis. While E. coli is not a predominant member of the oral microbiota, its LPS is widely used as a model molecule in inflammasome research due to its well-characterized structure and strong immunostimulatory properties [27]. Unlike species-specific variations in LPS structure, ultrapure E. coli LPS provides a standardized bacterial component to investigate fundamental inflammasome activation mechanisms. Furthermore, E. coli infection has commonly been detected in the oral cavity [32–34]. Given these factors, our findings on intracellular LPS-driven inflammasome activation are likely applicable beyond E. coli to other Gram-negative oral pathogens capable of delivering LPS intracellularly.

**Fig. 7:**
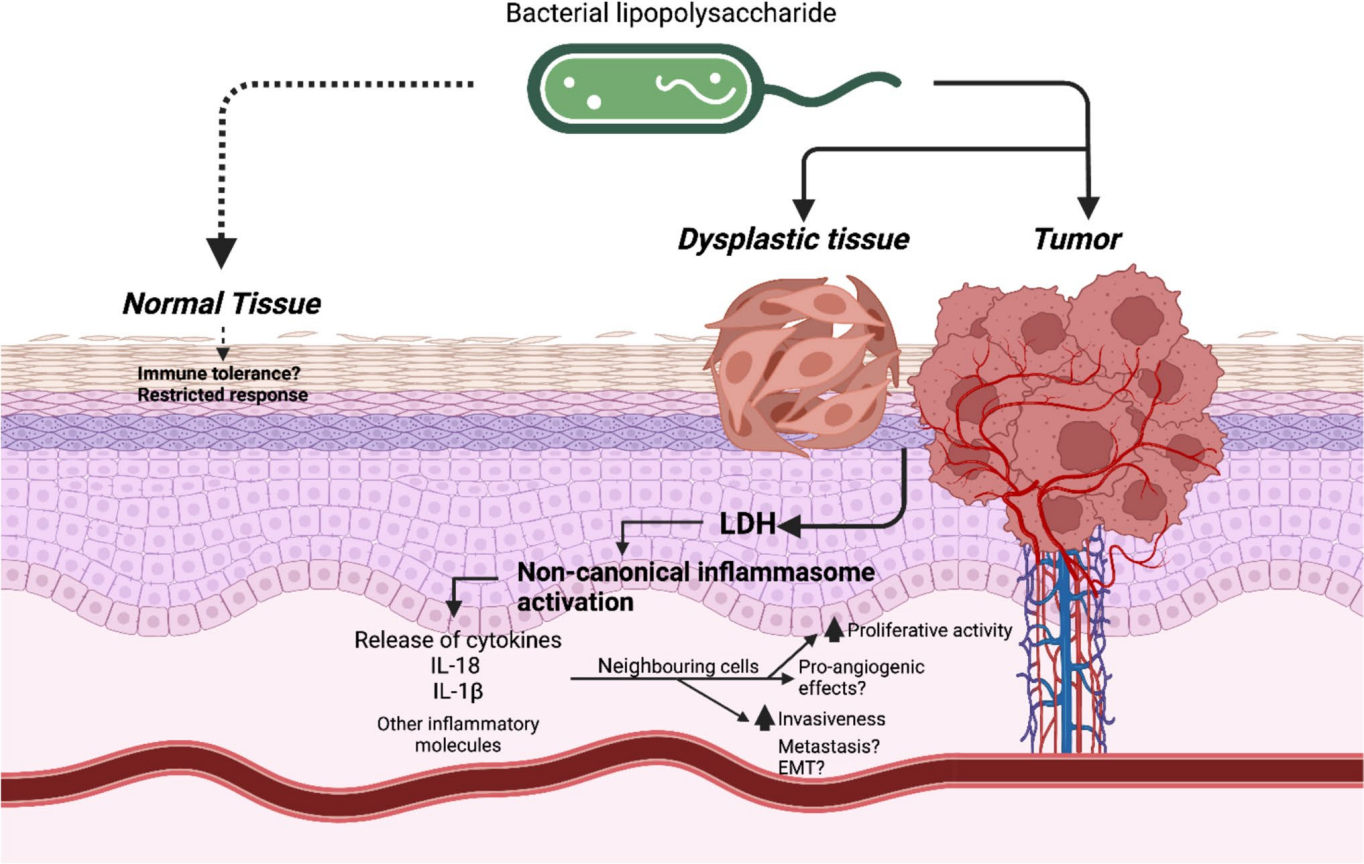
Schematic representation of the proposed role of bacterial LPS in oral carcinogenesis. Bacterial LPS is hypothesized to contribute to oral carcinogenesis through various mechanisms. Intracellular LPS triggers noncanonical inflammasome activation leading to pyroptotic cell death and the release of lactate dehydrogenase (LDH) accompanied by elevated secretion of the pro-inflammatory cytokines IL-18 and IL-1β from epithelial cells. These events may promote pro-tumorigenic effects in neighboring cells: angiogenesis, epithelial-mesenchymal transition (EMT), invasiveness and metastasis, thereby contributing to tumor progression

The apparent resistance of normal oral epithelial cells to noncanonical inflammasome-induced cytotoxicity may reflect robust homeostatic mechanisms that maintain epithelial integrity in the presence of commensal microbiota. Epithelial cells serve as the primary barrier and first line of defense against invading pathogens [35]. While healthy epithelium expresses Toll-like receptors (TLRs) capable of recognizing a wide range of microbial components, nasal epithelial cells were non-responsive to bacterial ligands including LPS [36, 37]. This restricted response promotes immune tolerance to commensal bacteria, thus maintaining homeostasis under steady-state conditions. Studies have shown that, unlike diseased epithelium, healthy epithelial cells require a ‘second signal’, such as IgG opsonization, to break immune tolerance and switch from a tolerogenic to an inflammatory phenotype [36, 38]. HPV-transformed and OSCC cells, characterized by disrupted homeostasis and altered cellular signaling, may lack these protective mechanisms, rendering them more susceptible to LPS-induced noncanonical inflammasome activation. This differential response highlights the potential of targeting bacterial components or inflammasome pathways in OSCC therapy. Elevated LDH levels in tumor tissues and serum have been associated with poor prognosis in several cancers, including melanoma, prostate, nasopharyngeal, and lung cancers [39]. Our observation of increased LDH release in HPV-transformed and OSCC cells upon LPS transfection aligns with these findings, suggesting that intracellular LPS may contribute to metabolic alterations that promote tumor progression (Fig. 7).

The activation of the noncanonical inflammasome pathway, as evidenced by the elevated production of IL-18 and IL-1β, further supports the pro-inflammatory impact of intracellular LPS in HPV-transformed and OSCC cells. Interestingly, SCC-24 A and SCC-24B cells robustly secreted IL-18 in response to intracellular LPS, while secretion of IL-1β remained minimal. This differential secretion pattern may stem from distinct regulatory mechanisms governing the availability of precursor cytokines. IL-18 is known to be constitutively expressed in many epithelial cells including oral keratinocytes, whereas IL-1β is not constitutively expressed under homeostasis [40, 41]. The weak IL-1β release in our model may thus reflect insufficient transcriptional priming or limited intracellular pools of pro–IL-1β, despite inflammasome activation. These observations are consistent with reports showing that IL-18 and IL-1β can be uncoupled in their expression and release dynamics [42]. Recent findings show that unlike caspase-11, caspase-4 can cleave IL-18 into its mature form, which may further affect the levels of secreted inflammasome-dependent cytokines in human [43]. Some of the responses observed in cells treated with extracellular LPS may be mediated through surface-expressed TLR4, activating canonical signaling pathways. Although we did not assess TLR4 expression directly, prior studies suggest that oral epithelial and carcinoma cells can express functional TLR4 under inflammatory conditions, which may contribute to LPS responsiveness [44, 45].

Both IL-18 and IL-1β are implicated in promoting the tumorigenesis of various cancers [46]. For instance, increased IL-18 levels have been reported in pancreatic ductal adenocarcinoma [47], gastric cancer [48], and cutaneous T-cell lymphoma [49], while IL-1β concentrations were elevated in the peripheral blood of patients with head and neck SCC [50], supporting the relevance of our findings in the context of cancer progression.

The selective induction of pyroptosis in HPV-transformed and OSCC cells may have significant implications for OSCC progression. Pyroptotic cell death leads to cell membrane rupture and the secretion of pro-inflammatory cytokines and cytoplasmic contents into the TME, potentially promoting inflammation-driven tumor growth and invasion [21]. While proliferation was assessed only in IHGK cells as a model of early transformation, further work is needed to evaluate whether intracellular LPS similarly affects proliferative behavior in malignant SCC cells. Our observation that factors secreted by metastatic cells following noncanonical inflammasome activation enhanced invasion in metastatic OSCC cell lines, but not in primary tumor cells, supports this notion. The increased invasiveness of metastatic cells suggests that noncanonical inflammasome activation may specifically promote aggressive phenotypes in cells that have already acquired certain malignant traits. Moreover, supernatants from metastatic cells activated with intracellular LPS also increased the invasion potential of primary tumor cells, indicating that noncanonical inflammasome activation can enhance the aggressiveness of neighboring cells through paracrine signaling. The enhanced invasiveness of SCC-24 A cells following exposure to pyroptotic supernatants may be driven by extracellular vesicles or soluble mediators released during inflammasome activation and subsequent pyroptosis. These factors may act in a paracrine fashion to activate signaling pathways in neighboring cells, promoting invasive or angiogenic phenotypes [21]. While this study did not directly identify the mediators responsible, the observation aligns with established models of inflammasome-driven tumor progression and warrants further investigation. This finding aligns with previous studies demonstrating that bacterial components can promote cancer cell invasion and metastasis. For instance, LPS has been shown to enhance invasiveness in breast cancer cells [51]. Additionally, LPS-induced angiogenesis, mediated through STAT3-dependent pathway, has been observed in hepatocellular carcinoma both in vitro and in vivo [52], which parallels our results showing increased tubulogenesis in metastatic cells following exposure to supernatants of intracellular LPS-activated cells. While this may reflect enhanced angiogenic potential, we acknowledge that increased cell proliferation may also contribute to the observed morphology. Tumor cell-derived VM is associated with poor prognosis and resistance to anti-angiogenic therapies [53, 54]. Our findings suggest that bacterial components within tumor cells can influence not only their invasive capacity but also their ability to adapt to hypoxic conditions, commonly associated with VM, and sustain tumor growth. Future studies using endothelial markers or time-lapse imaging would be necessary to confirm this phenomenon.

Although our model utilizes direct transfection of ultrapure LPS to ensure controlled intracellular delivery, it functionally mimics bacterial LPS entry mechanisms that have been described in other models. In particular, oral Gram-negative bacteria are known to release OMVs capable of fusing with epithelial cells and delivering LPS or other microbial components into the cytoplasm [55]. These processes parallel the findings of Nejman et al. [24], who demonstrated the presence of intracellular microbes in cancer cells. Thus, our approach offers a mechanistic approximation of these tumor-microbe interactions, focused specifically on intracellular LPS as a key immunomodulatory element. Further, our findings highlight the complexity of tumor biology, which can be reconciled by considering the dynamic and heterogeneous nature of cancer. In TME, it is plausible that only a subset of cancer cells undergo pyroptosis upon exposure to intracellular LPS, releasing inflammatory mediators that enhance the aggressiveness of neighboring viable cells [56]. Additionally, cancer cells may develop mechanisms to tolerate or exploit intracellular LPS. For instance, some cancer cells may upregulate anti-apoptotic pathways or modulate inflammasome activity to survive in a pro-inflammatory environment [57]. Furthermore, cancer stem cells could harness LPS to augment their aggressive phenotype, while regulatory immune cells within the TME may modulate the inflammatory response, promoting tumor progression [29, 58]. This model aligns with the concept that inflammation within the TME can be a double-edged sword, capable of both promoting and inhibiting tumor progression depending on the context [59].

Our findings resonate with emerging evidence that tumor-resident intracellular bacteria can actively shape cancer progression. For instance, Fu et al. demonstrated that intratumoral bacteria in breast cancer models promote metastatic colonization by enhancing tumor cell survival under fluid shear stress and reorganizing the actin cytoskeleton [22]. Although we focused on the noncanonical inflammasome pathway and subsequent pyroptosis triggered by intracellular LPS, these converging lines of evidence underscore that bacterial components—whether intact microbes or their derivatives—can fundamentally influence tumor behavior and metastatic potential in diverse cancer types. While our study provides mechanistic insights, in vitro models do not fully replicate the complexity of the TME, including interactions with immune cells, extracellular matrix components, and the microbiota. Future studies employing in vivo models with clinical cohorts are necessary to validate our findings and elucidate the mechanisms underlying the interplay between intracellular LPS and OSCC progression.

## Conclusion

Our findings suggest that intracellular bacterial LPS may not merely function as a passive intratumoral component but could potentially contribute to OSCC progression. By activating the noncanonical inflammasome, triggering pyroptosis, and amplifying pro-inflammatory cytokine secretion, LPS may influence a TME supportive of invasion and angiogenesis, particularly in metastatic settings. These findings broaden our understanding of the microbiota-tumor interactions in oral carcinogenesis and highlight a previously underappreciated therapeutic frontier. Targeting intracellular LPS or its downstream inflammasome pathways may represent a promising avenue to curtail tumor aggressiveness. Continued investigation in preclinical models will be essential to determine the translational relevance of these findings in OSCC.

## Supplementary Information

Below is the link to the electronic supplementary material.

Supplementary file1 (DOCX 119 KB)

## Data Availability

No datasets were generated or analysed during the current study.
