# Supplementary material for: Intracellular bacterial LPS drives pyroptosis and promotes aggressive phenotype in oral squamous cell carcinoma
Source: Med Oncol. 2025 May 8;42(6):205. doi: 10.1007/s12032-025-02766-6 (PMC12062154; doi:10.1007/s12032-025-02766-6)
Supplement: Supplementary file 1 — Supplementary file1 (DOCX 119 KB) [file 12032_2025_2766_MOESM1_ESM.docx]

**Intracellular bacterial LPS drives pyroptosis and promotes aggressive phenotype in oral squamous cell carcinoma**

Shrabon Hasnat^1^, Marjut Metsäniitty^1^, Katariina Nurmi^2^, Kari K. Eklund^2,3^, Abdelhakim Salem^1,2,*^

^1^Department of Oral and Maxillofacial Diseases, Clinicum, Faculty of Medicine, University of Helsinki, 00014 Helsinki, Finland

^2^Translational Immunology Research Program (TRIMM), Research Program Unit (RPU), University of Helsinki, 00014 Helsinki, Finland

^3^Department of Rheumatology, University of Helsinki and Helsinki University Hospital, Helsinki, 00014, Finland

^*^Correspondence to: Dr. Abdelhakim Salem

Head and Neck Oncobiome Group, Department of Oral and Maxillofacial Diseases, Clinicum

Faculty of Medicine, University of Helsinki, 00014 Helsinki, Finland

E-mail: abdelhakim.salem@helsinki.fi; Tel: +358 29 412 5237

**Supplementary figure**

**
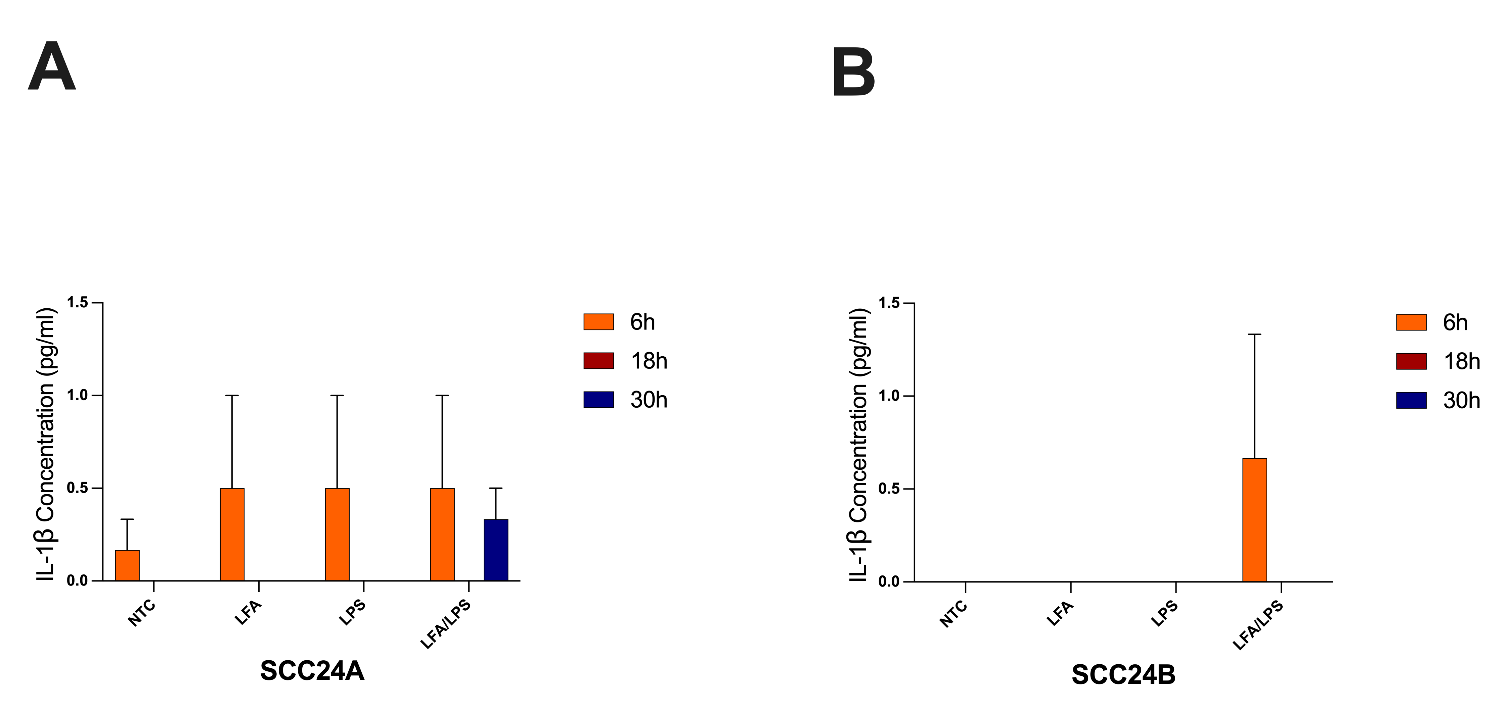
Supplementary Fig. 1** **Effect of intracellular LPS on the production of IL-1β across oral cancer cell lines.** Oral cancer SCC-24A and -24B cells were transfected with ultrapure LPS from *Escherichia coli* O111:B4 (2 µg/ml) using Lipofectamine 2000 (LFA), or with either LFA or LPS diluted directly in the medium for the indicated times to assess IL-1β secretion. These two cancer cell lines showed minimal IL-1β response.
